# Effect of conformation sampling strategies in genetic algorithm for multiple protein docking

**DOI:** 10.1186/1753-6561-6-S7-S4

**Published:** 2012-11-13

**Authors:** Juan Esquivel-Rodríguez, Daisuke Kihara

**Affiliations:** 1 Department of Computer Science, College of Science, Purdue University, West Lafayette, IN 47907, USA; 2 Department of Biological Sciences, College of Science, Purdue University, West Lafayette, IN 47907, USA

## Abstract

**Background:**

Macromolecular protein complexes play important roles in a cell and their tertiary structure can help understand key biological processes of their functions. Multiple protein docking is a valuable computational tool for providing structure information of multimeric protein complexes. In a previous study we developed and implemented an algorithm for this purpose, named Multi-LZerD. This method represents a conformation of a multimeric protein complex as a graph, where nodes denote subunits and each edge connecting nodes denotes a pairwise docking conformation of the two subunits. Multi-LZerD employs a genetic algorithm to sample different topologies of the graph and pairwise transformations between subunits, seeking for the conformation of the optimal (lowest) energy. In this study we explore different configurations of the genetic algorithm, namely, the population size, whether to include a crossover operation, as well as the threshold for structural clustering, to find the optimal experimental setup.

**Methods:**

Multi-LZerD was executed to predict the structures of three multimeric protein complexes, using different population sizes, clustering thresholds, and configurations of mutation and crossover. We analyzed the impact of varying these parameters on the computational time and the prediction accuracy.

**Results and conclusions:**

Given that computational resources is a key for handling complexes with a large number of subunits and also for computing a large number of protein complexes in a genome-scale study, finding a proper setting for sampling the conformation space is of the utmost importance. Our results show that an excessive sampling of the conformational space by increasing the population size or by introducing the crossover operation is not necessary for improving accuracy for predicting structures of small complexes. The clustering is effective in reducing redundant pairwise predictions, which leads to successful identification of near-native conformations.

## Background

The tertiary structure of proteins provides valuable information about the mechanisms of protein function, however, structures of multimeric protein complexes are often difficult to solve by experimental methods. Even in the cases that the structure of an entire complex has not been determined, the structure of the individual subunits are often available, either because they have been solved experimentally or computationally modeled. Multi-LZerD is a multiple protein docking protocol developed by our group [1-3], which takes structures of individual subunits and assembles them into complex models. The method was shown to be able to construct near-native structures both for bound and unbound docking cases. It was shown Multi-LZerD achieved overall better performance than a competitive method especially in unbound docking of multiple subunits [3].

**Figure 1:**
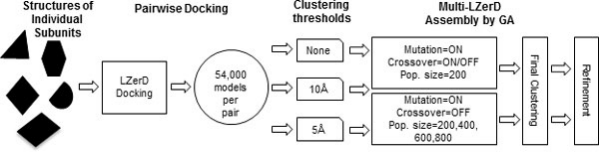
**Overview of Multi-LZerD and this experiment**. Multi-LZerD builds a protein complex by assembling pairwise docking predictions computed by LZerD. For each pair 54,000 models will be generated. The pairwise predictions can be clustered using a RMSD cutoff value of 5 or 10 Å or used for assembly without clustering. Pairwise docking models are assembled using GA. We tested different settings of the GA runs, with/without crossover operation, and different population sizes. After the final generation of GA, models can be clustered and refined, which we did not perform in this work.

Multi-LZerD is composed of two main stages (Figure 1). First, we compute pairwise docking predictions between all pairs of subunits by mainly considering shape complementarity of the subunits [4]. In the second stage, we represent entire multimeric complex structures using graphs where nodes denote subunits and the edges specify a pairwise transformation between subunits, which are computed in the pairwise docking stage. At the beginning of the second stage, a configurable number of random graphs are created to explore different graph topologies. Pairwise transformations are randomly selected from the pool of pairwise docking solutions computed in the first stage. The population of graphs is iteratively improved in terms of a fitness function using a Genetic Algorithm (GA), by exploring different topologies and pairwise transformations for edges. The fitness function is a linear combination of physics-based and/or knowledge-based scoring terms including van der Waals potential, electrostatic potential, and a knowledge-based atom contact potential [3] (i.e. not just shape matching as used in the first stage). At each generation, models are clustered to remove redundancy in the population.

As it is typical for stochastic evolutionary optimization approaches, the parameters used to execute the simulations affect the accuracy of the final results as well as the computational cost. In particular, we would like to optimize the number of random graphs used as the initial input and those kept at each GA generation (the population size) as well as the population variability given by the mutation and the crossover operators. Using these two operators increases the population size temporarily, before selecting the best fitted structures that are passed to the subsequent generation. The computation of a physics-oriented fitness function can have a significant cost if the population size is too large. Oppositely, if the population size is too small, it may require a large number of generations to find a near-native model. We also tested different pairwise docking clustering thresholds. The pairwise docking decoys for each pair of subunits are clustered based on the root mean square deviation (RMSD) of Cα atoms. The procedure implemented is based on the clustering ideas conceived in ClusPro [5]. Two complexes are considered to be neighbors if they are closer than a threshold value. Once the clusters are created, the decoy with the best shape-based score is selected out of each cluster as a representative structure. The other members in the clusters will be deleted.

In this work, we analyzed the effect of the three parameters in the GA optimization in Multi-LZerD, namely, the population size, whether to use the crossover operation, and the threshold value in the structural clustering (Figure 1). The results suggest that an excessive sampling of the conformational space is not necessary in our multiple-docking procedure to find correct structure models.

## Methods

We assessed the effect of different GA parameters used in Multi-LZerD on the computational time and the accuracy. The original Multi-LZerD is configured as follows: Given a set of tertiary structures of subunits of a protein complex, the pairwise protein docking algorithm, LZerD [4], is run to produce 54,000 docking candidates (decoys). A conformation of a whole protein complex is uniquely defined by a spanning tree (graph) where each node is connected to at least another node by an edge. This is suitable for constructing a multiple docking complex from pairwise decoys because not all pairs of nodes need to be connected. An edge between a pair of subunits specifies one of 54,000 pairwise docking decoys. Starting with *M *spanning trees, with *M *being 200 in the original setting, various alternative conformations are generated by GA with mutation or crossover operations. The mutation deletes one of the spanning tree edges and then selects a new edge randomly to reconnect the graph. It is possible that the same edge is selected again. Then, for a newly selected edge, one of the pairwise docking decoys for the two subunits is randomly selected. The rest of the edges remain unaltered. The crossover takes two candidate structures in the current population and creates a new individual by combining edges from the two parents. It will first create an empty graph and randomly select edges from the parents until a spanning tree is created. The decoys are subject to clustering with a predefined threshold value of RMSD. Finally, *M *= 200 decoys with the best fitness scores are selected for the next generation. In the final generation, clustering will yield at most *M *= 200 decoys ranked by their fitness score as the final prediction.

Two experiments were performed (Figure 1). The first experiment was to examine the effect of using the crossover operation together with the mutation operator in the GA. We executed Multi-LZerD in two different settings:

1) Enable both mutation and crossover operations. Decoys of a population size of *M *(200) were subject to the application of the crossover operation, which was set to increase the population by 50% (i.e. 300). Then, the mutation operation is applied, which was configured to double the population size (i.e. 300 × 2 = 600). The 600 decoys were clustered and ranked by the fitness score, and the top 200 decoys were passed to the next iteration.

2) Enable mutation and disable crossover. For a population of 200 decoys, the mutation operation increases it to 400, which were subject to the clustering and the selection by the fitness score.

In both 1) and 2), the within-generation clustering was performed using a threshold value of 10 Å.

The second experiment is to examine the effect of different population sizes, 200, 400, 600, and 800 at each GA generation. Obviously, increasing the population size is costly but at the same time near native models may be found at an earlier GA generation. In this experiment, only the mutation operation was used and the threshold of the within-generation clustering was set to 10 Å.

We have also examined different clustering cutoffs for pairwise docking decoys computed by LZerD. Three pairwise clustering settings were tested, using either 5 Å or 10 Å Cα RMSD, or without using clustering (Figure 1). For the last setting, all 54,000 pairwise predictions generated by LZerD were used. The three clustering settings are tested in combination with the four different population sizes.

## Results

**Figure 2:**
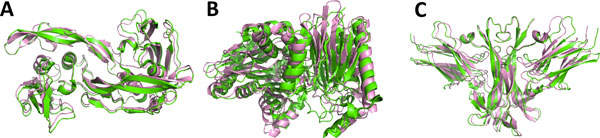
**Best models obtained for each test case**. The best model from all the experiments is shown for each of the three complexes. **A**, a 1.14 Å RMSD model for 1ES7; **B**, the best model for 1K2X, RMSD: 2.12 Å; **C**, a 2.48 Å RMSD model for 1WWW. The native structure is shown in green while the prediction is shown in pink.

The above experiments were performed on three protein complexes, BMP-2-BRIA ectodomain complex (PDB ID: 1ES7 ), plant-type L-asparaginase (1K2X), and nerve growth factor/trka complex (1WWW). All three complexes consist of four chains. Figure 2 shows the native structure of the three protein complexes superimposed with the best models obtained in the experiments.

### Impact of using the crossover operator

**Figure 3:**
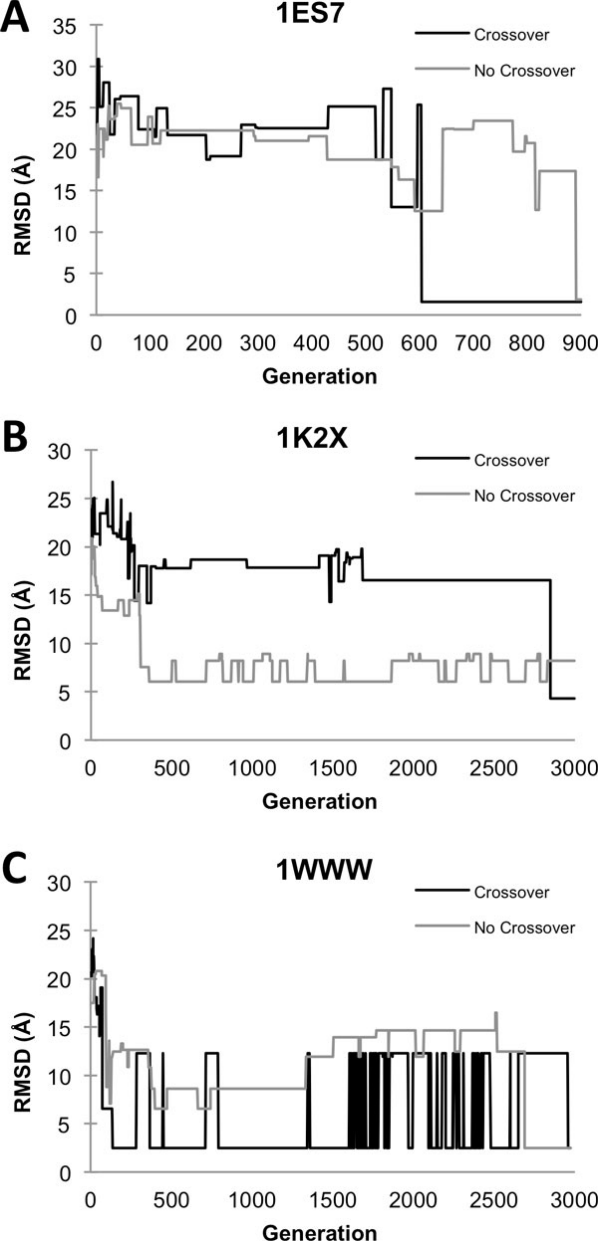
**Improvement of RMSD of the best model along GA generations**. For three protein complexes, A, 1ES7; B, 1K2X; C, 1WWW, GA was applied with and without crossover starting with a population size of 200. Black lines represent the GA runs where both crossover and mutation were applied, while gray lines show results of the mutation-only conformation search.

Figure 3 shows the progressive improvement of the RMSD of the best model at each GA generation using both crossover and mutation, as well as the mutation-only settings. In the case of 1ES7 (Figure 3A) and 1WWW (Figure 3C), both settings yielded near-native predictions (an RMSD of 2.5 Å or less to the native). For 1K2X (Figure 3B) the best RMSD obtained with crossover is 4.30 Å, while the run with mutation-only yielded a model with 8.21 Å.

In the case of 1ES7, the GA run with crossover generated a near native structure at an earlier generation of around 600. In contrast, the GA without crossover found a near native structure at around the 900^th ^generation. Although the GA with crossover found the near-native structure earlier, the actual computational cost was similar because using the crossover increases the population size at each generation by 1.5 times, which increases the clustering cost by 1.5^2 ^= 2.25 times, as we will discuss in the computational cost section. In the next case (Figure 3B), the GA run without crossover quickly found the structures between 6-8 Å RMSD, starting with 7.53 Å at the 308th generation. However, the GA with crossover found a better structure at the end around the 3000^th ^generation. In the last example (Figure 3C), the GA with crossover identified a near native structure at a significantly earlier generation (136th) than without crossover, which yielded a near-native structure at the 2689th.

Overall, the GA with crossover found a near-native structure at an earlier generation for two out of three cases. However, in one case (1ES7, Figure 3A) the actual computational cost for the GA with and without crossover was comparable.

### Impact of the population size and the clustering threshold

Next, we tested four different population sizes (200, 400, 600, and 800) and three different pairwise clustering settings (10 Å, 5 Å, and not using the clustering step). Table 1 shows the summary of the best model (i.e. the model with the smallest RMSD) obtained by using GA runs for each setting. In addition to the RMSD of the best model, the rank of the fitness score, and the fnat, the fraction of native contacts [6], are also shown.

**Table 1:** Summary of predictions using different population sizes and clustering thresholds

		No Pairwise Clusters			5Å Pairwise Clusters			10Å Pairwise Clusters		
PDB	Population Size	RMSD (Å)^1^	fnat^2^	Rank	RMSD (Å)^1^	fnat^2^	Rank	RMSD (Å)^1^	fnat^2^	Rank
1ES7	200	17.51	0.03	20	17.94	0.03	16	1.86	0.92	3
	400	9.85	0.25	159	7.95	0.63	47	1.86	0.92	3
	600	14.21	0.00	445	9.56	0.34	265	1.86	0.92	3
	800	2.15	0.70	18	7.23	0.64	2	1.86	0.92	3

1K2X	200	18.52	0.00	84	21.20	0.01	61	6.05	0.49	74
	400	20.36	0.01	239	6.05	0.49	80	23.69	0.01	195
	600	6.14	0.46	250	2.12	0.51	1	23.41	0.01	599
	800	19.45	0.01	56	19.26	0.02	718	6.04	0.49	60

1WWW	200	19.16	0.02	51	13.49	0.11	152	8.62	0.58	23
	400	5.34	0.52	10	15.58	0.00	115	2.48	0.78	12
	600	17.37	0.03	248	15.76	0.03	182	11.12	0.39	13
	800	16.30	0.00	160	17.19	0.02	615	2.48	0.78	14

GA runs were carried out for 1000 generations. The crossover operation was not used.

^1^The Global C-α RMSD between the best prediction in the final population and the native structure, for each simulation.

^2^fnat is the fraction of correctly predicted interface residues.

The first observation is that results using the 10 Å cutoff showed the best RMSD models in ten out of twelve cases as compared with results without clustering and with the 5 Å cutoff. Comparing the no clustering and the 5 Å cutoff, the latter performed better than the former for seven out of twelve cases. The results indicate a better result can be expected in general when a larger cutoff is used for clustering for reducing redundancy to efficiently explore the conformational space.

As for the population size, we did not observe a clear trend relative to the best RMSD. Thus, we temporarily conclude that the population size of 200 or 400 is sufficient for the multiple docking by Multi-LZerD for the complexes of four chains. Although we did not observe improvement of prediction accuracy by increasing the population size for the current dataset, a larger population size may work better for complexes of a larger number of chains.

### Computational cost

The computational time of the GA optimization in Multi-LZerD consists mainly of the pairwise docking by LZerD along with the pairwise clustering step, and the followings at each GA generation: the calculation of the fitness score for each decoy, the mutation operation for each decoy, the crossover operation for pairs of decoys, and the clustering step performed at each generation. The time complexity of computing the fitness score, the mutation operation, and the crossover operation is linear in the number of decoys, while it is quadratic for the clustering step because it needs pairwise RMSD values between all decoy pairs in the population. Thus, the overall time complexity is quadratic in the number of decoys in the population. The use of the crossover operation has a significant impact in the overall computational time because it will increase the number of decoys in the population that are subject to the clustering.

**Figure 4:**
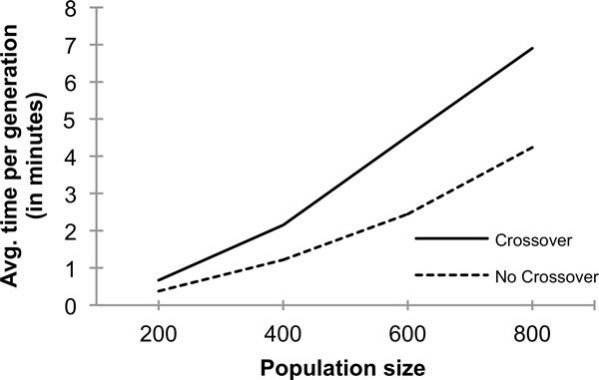
**Computational time of running Multi-LZerD**. Average running time per generation (in minutes) required by Multi-LZerD. Four different population sizes, 200, 400, 600, and 800 were tested with and without using the crossover operation. A protein complex, 1ES7, was used. Multi-LZerD was executed on 10 Intel Xeon L5630 CPUs at 2.13GHz. The computational time on the y-axis is the average of 25 generations.

In Figure 4, we show the actual average computational time of one Multi-LZerD GA generation, for the protein complex 1ES7, with and without the crossover operation. In both cases, the computational time roughly grows in a quadratic fashion as the population size grows. Comparing the time of running Multi-LZerD with and without crossover at each population size, the time using the crossover is about 1.6 to 1.8 times larger than that of without using crossover. As described above, when the initial population size is *2N*, applying the mutation increases the population to *4N *while using both crossover and mutation operations increases it to *6N*. Thus, the quadratic computational cost for the clustering step for the latter will take (*6N*/*4N*)^2 ^= 1.5^2 ^= 2.25 times more than the former. The observed increase of the population size in Figure 4 roughly agrees with this estimation.

## Conclusions

Multi-LZerD employs GA for exploring the conformation space and there are several key parameters that can critically affect the prediction performance. Since computing the fitness score is a time-consuming step, it is desired to keep the population of decoys in each GA generation small, but at the same time the algorithm should be able to explore the conformation space sufficiently to find near-native models. From the testing of Multi-LZerD on the three protein complexes, we found that the population size of 200 or 400 is sufficient (Table 1). The clustering is effective in reducing redundant pairwise predictions, which leads to successful identification of near-native conformations. Using the crossover operation yielded a near-native structure in an earlier generation than without using the crossover; however, they yielded similar final prediction results after a larger number of iterations. The current study leads to the conclusion that an excessive sampling of the conformational space is not necessary for small protein complexes (around four subunits) to find correct structure models in the Multi-LZerD scheme. However, it is not clear if the conclusion applies to complexes with a larger number of subunits since their conformational space can be significantly larger than smaller ones.

## List of abbreviations used

• GA: Genetic Algorithm; • fnat: Fraction of native contacts; • PDB: Protein Data Bank; • RMSD: Root mean square deviation.

## Competing interests

The authors declare that they have no competing interests.

## Authors' contributions

JER developed the multiple protein docking prediction method, Multi-LZerD, performed the computational experiments and wrote the manuscript draft. DK conceived the study and participated in its design and coordination, as well as drafting and finalizing the manuscript. All authors read and approved the final manuscript.
